# Investigating the role of aae-miR-34-5p in the regulation of juvenile hormone biosynthesis genes in the mosquito *Aedes aegypti*

**DOI:** 10.1038/s41598-023-46154-4

**Published:** 2023-11-03

**Authors:** Mazhar Hussain, Zhi Qi, Lauren M. Hedges, Marcela Nouzova, Fernando G. Noriega, Sassan Asgari

**Affiliations:** 1 Australian Infectious Disease Research Centre, School of Biological Sciences, The University of Queensland, Brisbane, QLD Australia; 2 Institute of Parasitology, Biology Centre CAS, České Budějovice, Czech Republic; 3 Department of Biological Sciences and Biomolecular Sciences Institute, Florida International University, Miami, FL USA; 4 Department of Parasitology, University of South Bohemia, České Budějovice, Czech Republic

**Keywords:** Entomology, miRNAs

## Abstract

Juvenile hormone (JH) controls the development and reproduction of insects. Therefore, a tight regulation of the expression of JH biosynthetic enzymes is critical. microRNAs (miRNAs) play significant roles in the post-transcriptional regulation of gene expression by interacting with complementary sequences in target genes. Previously, we reported that several miRNAs were differentially expressed during three developmental stages of *Aedes aegypti* mosquitoes with different JH levels (no JH, high JH, and low JH). One of these miRNAs was aae-miR-34-5p. In this study, we identified the presence of potential target sequences of aae-miR-34-5p in the transcripts of some genes encoding JH biosynthetic enzymes. We analysed the developmental expression patterns of aae-miR-34-5p and the predicted target genes involved in JH biogenesis. Increases in miRNA abundance were followed, with a delay, by decreases in transcript levels of target genes. Application of an inhibitor and a mimic of aae-miR-34-5p led respectively to increased and decreased levels of thiolase transcripts, which is one of the early genes of JH biosynthesis. Female adult mosquitoes injected with an aae-miR-34-5p inhibitor exhibited significantly increased transcript levels of three genes encoding JH biosynthetic enzymes, *acetoacetyl-CoA thiolase* (thiolase), *farnesyl diphosphate phosphatase*, and *farnesal dehydrogenase*. Overall, our results suggest a potential role of miRNAs in JH production by directly targeting genes involved in its biosynthesis.

## Introduction

Juvenile hormone (JH) plays major roles in the control of mosquito development and reproduction^1–3^. JH synthesis stops in the late larval stage and resumes late in the pupal stage. This increase in JH titre in newly emerged adult females plays a key role in ovarian maturation. Following eclosion, the JH titre remains high in sugar-fed females, but drops sharply after blood feeding. This sequence is repeated in each gonotrophic cycle (reviewed in^3^).

JH is synthesized in the *corpora allata* (CA), a pair of small endocrine glands connected to the brain^4^. The JH biosynthetic pathway involves 13 enzymatic steps occurring in a sequential order^5^. Previous studies have shown that the genes coding for the enzymes are coordinately expressed in the *Aedes aegypti* mosquito; as well as a correlation between the expression of these genes and the JH levels^5^. Therefore, tight regulation of the expression of these genes during different developmental and reproductive stages is important.

microRNAs (miRNAs) play significant roles in the post-transcriptional regulation of gene expression by interacting with complementary sequences in the target genes^6^. miRNAs participate in the regulation of nearly all cellular process investigated so far, and their dysregulation is often associated with developmental and reproductive disorders^7^. In insects, miRNAs are involved in many biological processes, including development, behaviour, immunity, and host–pathogen interactions (reviewed in^8,9^). Previously, we reported differential expression of miRNAs in CA glands from three different developmental stages of the female *Ae. aegypti* with different levels of JH titre; early pupae (nearly no JH synthesis), sugar-fed adults (high JH synthesis), and blood-fed adults (very low JH synthesis)^10^. Five major groups of miRNAs were found in the three different developmental stages, representing distinct expression patterns. A number of miRNAs were specific to pupae (Group II), and some were specific for adults (Group IV and V); with some expressed only in blood-fed females (group IV) or were absent only after a blood meal (Group I). Some miRNAs were present only in pupae and blood-fed females that had low levels of JH synthesis (Group III)^10^.

In our previous study, aae-miR-34-5p was identified as one of the 20 most abundant miRNAs in *Ae. aegypti* CA, with higher abundance in blood-fed mosquitoes when compared to pupae and sugar-fed mosquitoes. Several genes coding neuropeptides or JH biosynthetic enzymes were predicted to be targets of this miRNA^10^. In this follow-up study, we used in vivo experiments and cell transfection assays to investigate aae-miR-34-5p interactions with target sequences in the transcripts of JH biosynthetic genes. In addition, we analysed the developmental expression patterns of aae-miR-34-5p and the predicted target genes involved in JH biogenesis. The results of these studies suggest that miRNAs could modulate JH synthesis by targeting genes encoding some of the biosynthetic enzymes.

## Materials and methods

### Mosquito and cell culture

*Aedes aegypti* mosquitoes (Innisfail) were maintained at 28 °C, 65–70% relative humidity, and 12 h light/12 h dark cycling regime. Larvae were fed on Hikari Cichlid Gold pellets, and adult mosquitoes on 10% sugar water ad libitum. Female mosquitoes were artificially fed human blood obtained from the Australian Red Cross (UQ ethics HE000850) equilibrated to 37 °C using glass feeders.

*Ae. aegypti* Aag2 cells were maintained at 27 °C in a 1:1 mixture of Mitsuhashi–Maramorosch (HIMEDIA, cat# IML002) and Schneider’s Drosophila Medium (Invitrogen, cat# 21720024), supplemented with 10% fetal bovine serum (Interpath, cat# AUFBS/PG DIP).

Sf9 cells (derived from *Spodoptera frugiperda*) were grown in SF900II medium (Gibco, ThermoFisher Scientific, cat# 10,902,096) as monolayer in flasks at 27 °C. Sf9 were used for miRNA-target interactions studies (see below).

### RNA extraction and reverse transcription quantitative PCR (RT-qPCR)

RNA was extracted from cells or individual mosquitoes using Qiazol (QIAGEN, cat# 79306). Extracted RNA was treated with DNase using TURBO DNase (Invitrogen, Thermo Fisher Scientific, cat# AM2238). cDNA was synthesised with a M-MuLV reverse transcriptase kit (New England BioLabs, cat# M0253L) using oligo-dT primer according to the manufacturer’s instructions. Quantitative PCR (qPCR) was performed in two technical replicates using QuantiFast SYBR Green PCR Kit (QIAGEN, cat# 204056) in a Qiagen Rotor-Gene Q under the following conditions: 95 °C for 30 s, and 40 cycles of 95 °C for 10 s and 60 °C for 45 s. The qPCR run was followed by a melting curve analysis (68–95 °C). For mosquito cells and adults, *ribosomal protein S17* (*RPS17*), and for *Spodoptera frugiperda* Sf9 cells, which were used for miRNA-target validation studies (see below), *actin* were used as normalising genes.

For quantification of aae-miR-34-5p, small RNAs were reverse transcribed with miScript II RT kit (QIAGEN, cat# 218160) using the HiSpec buffer with 250 ng of total RNA per sample according to the manufacturer’s instructions. qPCR was performed with a miScript SYBR Green PCR kit (QIAGEN, cat# 218161) in a QIAGEN Rotor-Gene Q using 10 times dilution of cDNA per reaction. 5s rRNA was used for normalising the data.

### The expression patterns of aae-miR-34-5p and the JH biosynthetic genes

To assess the expression patterns of aae-miR-34-5p and the JH biosynthetic genes, different developmental stages of female *Ae. aegypti* mosquitoes were collected before and after adult emergence; namely, 2 and 1 days before adult emergence, and 1–4 days after adult emergence. Total RNA was extracted from individual mosquitoes as described above, and then subjected to RT-qPCR analysis of aae-miR-34-5p and the JH biosynthetic genes as described above.

### miRNA-target validation

To validate miRNA-target interactions, predicted target sequences of aae-miR-34-5p in *Ae. aegypti acetoacetyl-CoA thiolase* (Thiolase), *farnesyl diphosphate phosphatase* (FPPase), and *farnesal dehydrogenase* (FALDH) (2 target sequences) were cloned downstream of the *GFP* gene in the pIZ/V5-His insect plasmid vector. Following confirmation of sequences by Sanger sequencing, the plasmids and the aae-miR-34-5p mimic or a negative control mimic (scrambled unrelated sequences, Table S1) were co-transfected into Sf9 cells (derived from *Spodoptera frugiperda*), which were grown in SF900II medium (Gibco, ThermoFisher Scientific, cat# 10902096), using Cellfectin transfection reagent according to the manufacturer’s instructions (Invitrogen, ThermoFisher Scientific, cat# 10362100). It is common to use heterologous insect cell lines for miRNA-target interactions to reduce the effect of endogenous miRNAs on target sequences. Three days after transfection cells were collected, RNA was extracted and subjected to RT-qPCR using specific primers to GFP (Table S1) to assess the expression levels of *GFP*. The *S. frugiperda actin* gene was used as the reference gene. If there is an interaction between the target sequences and the miRNA, a change in the expression levels of *GFP* can be expected.

To assess the effect of miRNA manipulation on thiolase expression in mosquito cells, Aag2 cells were transfected with aae-miR-34-5p mimic or inhibitor or their corresponding negative controls (scrambled unrelated sequences, Table S1). miRNA mimics/inhibitors and their controls were produced by Genepharma. About 500,000 cells were seeded per well in a 12-well plate and allowed to settle for 1h. Cells were then transfected with 100 nM of mimic or inhibitor per well using Cellfectin transfection reagent according to the manufacturer’s instructions (Invitrogen, ThermoFisher Scientific, cat# 10362100). Cells were then collected three days after transfection for RNA extraction and subsequent RT-qPCR analysis as described above.

### Mosquito injections and fecundity measurements

For injections of miRNA inhibitors/mimics, 1-day-old female mosquitoes were chilled on ice for 5 min. Using a Nanojet III (Drummond) and pulled glass needles, 125 nL of 200 μM solution of aae-miR-34-5p inhibitor/mimic or control inhibitor/mimic in *Aedes* physiological solution (APS; 150 mM sodium chloride, 4 mM potassium chloride, 0.1 mM sodium bicarbonate, 0.6 mM magnesium chloride, 1.7 mM calcium chloride, 25 mM HEPES Buffer at a pH of 7.0) were injected into the thorax of anaesthetized mosquitoes. Three days after injection, mosquitoes were fed on human blood using an artificial feeder. After feeding, 2–7 mosquitoes were pooled in the same container. The eggs laid per container were counted, and then averaged by the number of mosquitoes. In a replicate experiment, mosquitoes were kept individually in small cups following blood feeding. Eggs laid, hatching rates, and larvae per female were then scored.

For the assessment of responses of target genes to miR-34 inhibition, we injected 2–3-day-old females as described above, and analysed them 4-days post injection. Considering the CA is a very small gland located in the first thoracic segment, we dissected the head and first thoracic segment from each mosquito, and extracted RNA individually. The RNA samples were subjected to RT-qPCR analysis as described above.

### Uptake of small RNAs by the* corpora allata*

Four-day-old female *Ae. aegypti* mosquitoes were dissected to remove their *corpora allata*-*corpora cardiaca* (CA-CC). Dissected heads with CA-CC were incubated in 48-well plates for 4 h at 33 °C in 240 $\mu$ l of M-199 medium with 100nM Block-it AlexaFluor red fluorescent control (Thermo Fisher scientific, catalogue number 14750–100) as described above. The glands were observed and photographed using a DM 5500 B Leica fluorescence microscope with a Leica DFC 310 FX mounted camera and Leica LAS imaging software.

### Statistical analyses

All statistical analyses were performed with GraphPad Prism. T-test (validation of direct miRNA-target interactions, Fig. 2; confirmation of overexpression and knockdown of miR-34, Fig. 5A) or One-way ANOVA (all the rest of the experiments) were performed if the number of samples were two, or three or more, respectively. For RT-qPCR data analysis, the relative expression ratio method was used as described previously^11^. Gene expression levels in controls were adjusted to 1, and the transcript levels in treatments are expressed as fold changes relative to the controls.

## Results

### Target sequences of miR-34 in the transcripts of JH biosynthetic genes

**Figure 1:**
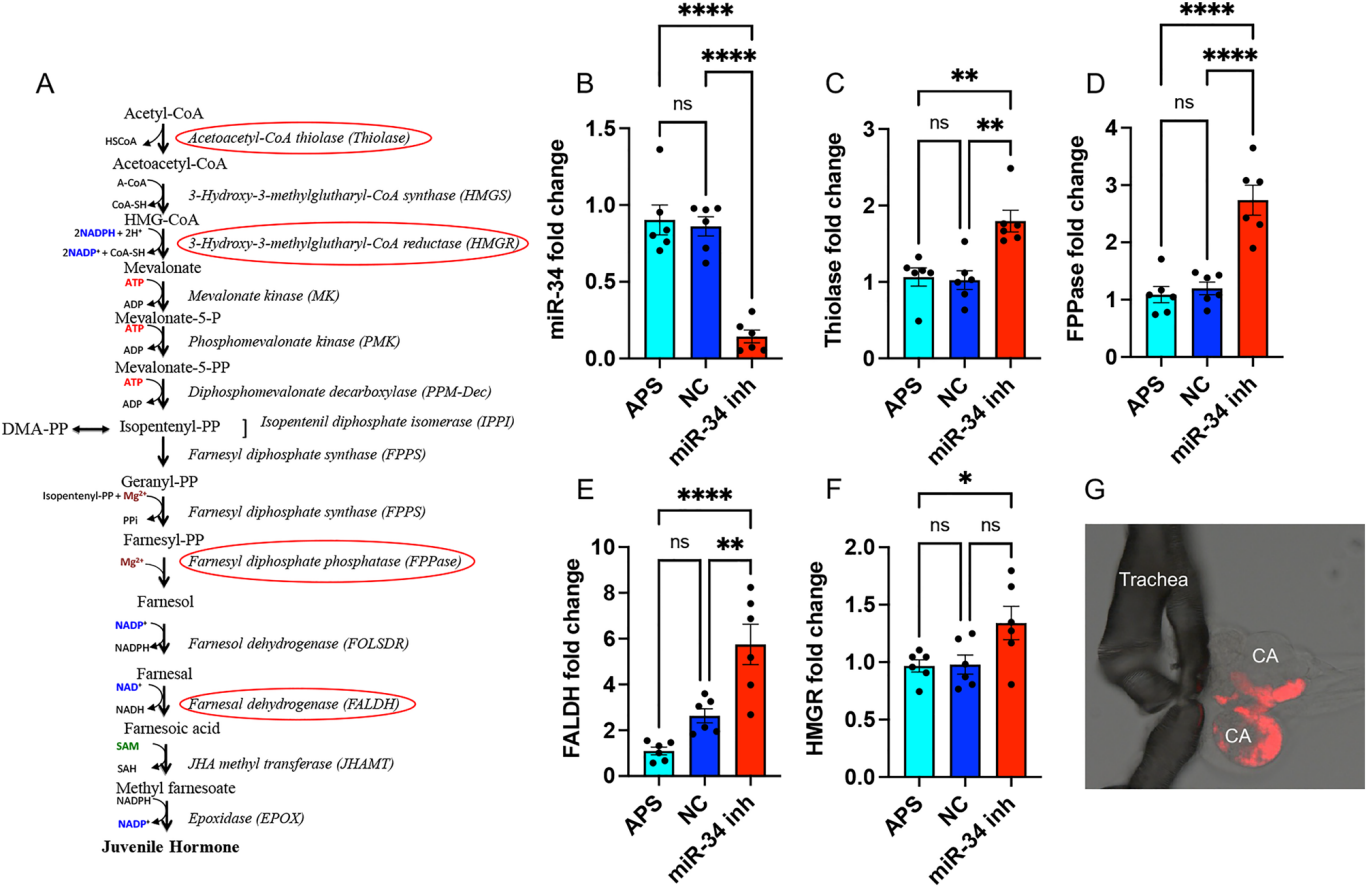
The effect of miR-34 inhibitor on the expression of JH biosynthetic genes. (**A**) JH biosynthesis pathway modified from ^30^. The red circled enzymes are potential targets of miR-34. (**B**) The expression levels of miR-34 in the head + first thoracic segment of *Ae. aegypti* mosquitoes four days after injection with buffer (APS), negative control (NC) inhibitor, and miR-34 inhibitor assessed by RT-qPCR. The inhibition of miR-34 was confirmed. (**C**–**F**) The relative expression levels of four target genes in the JH biosynthetic pathway (red circled in A) in the head + first thoracic segment of *Ae. aegypti* mosquitoes treated as in (B) assessed by RT-qPCR. One-way ANOVA with Tukey’s post hoc analysis was used to compare the treatments. The error bars represent standard error of mean (SEM) of biological replicates each represented by a data point. ns, not significant; *, *p* < 0.05; **, *p* < 0.01; ****, *p* < 0.0001. (**G**) Fluorescent microscopy of CA incubated with a small RNA conjugated with Alexa Fluor 555.

We investigated the presence of potential target sequences of miR-34-5p (we refer to it as miR-34 hereafter) in the transcripts of JH biosynthetic genes. RNAHybrid revealed potential target sequences with sequence complementarities to miR-34 in four genes (Fig. 1A). We found three target sequences in *3-Hydroxy-3-methylglutharyl-CoA-synthase* (*HMGR*), two in *Farnesal dehydrogenase* (*FALDH*), four in *Acetoacetyl-CoA thiolase* (*Thiolase*), and one in *Farnesyl diphosphate phosphatase* (*FPPase*) transcripts (Table S2).

To find out if these genes are regulated by miR-34, 2–3-day-old female mosquitoes were injected with buffer only (APS), a negative control inhibitor (NC), or a miR-34 inhibitor, and collected four days post-injection. We dissected head + first thoracic segment from individual mosquitoes. RNA was extracted and subjected to RT-qPCR analysis. First, the inhibition of miR-34 was confirmed in the mosquitoes (84% reduction; Fig. 1B). RT-qPCR analysis of RNA from the samples using specific primers for the four genes showed that in the case of *Thiolase*, *FPPase*, and *FALDH* inhibition of miR-34 led to significant increased transcript levels of the genes (Fig. 1C,E). However, in the case of *HMGR*, while inhibition of the miRNA led to an increase in the abundance of its transcript levels, it was not statistically significant when compared to both controls (Fig. 1F).

We used the head + first thoracic segment of mosquitoes to examine whether the predicted target genes respond in vivo to the miR-34 inhibitor. As a proof of concept, to confirm whether small RNAs are taken up by the CA, CA-CC glands were dissected from 4-day-old mosquitoes and incubated with a small RNA conjugated with Alexa Fluor 555. Fluorescent microscopy confirmed the presence of the labelled small RNA inside the CA glands after 4 h of incubation (Fig. 1G).

### In vitro validation of target genes

**Figure 2:**
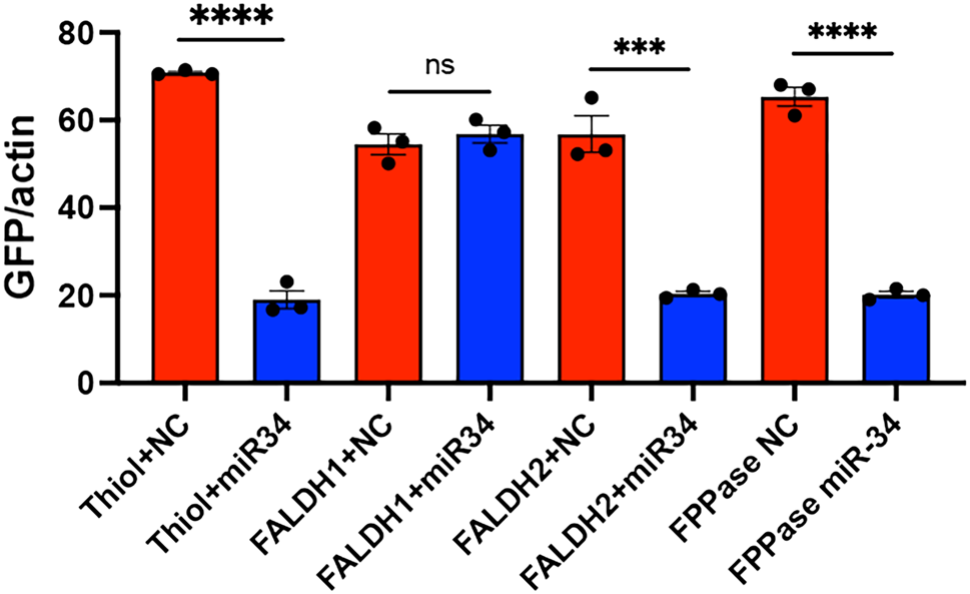
Assessment of direct interactions of miR-34 with the predicted target sites in *Thiolase*, *FALDH* (2 sites), and *FPPase*. The target sites were cloned downstream of the *GFP* gene (Fig. S1), and co-transfected with miR-34 mimic or the negative control (NC) mimic into Sf9 cells. *GFP* transcript levels were assessed two days after transfection by RT-qPCR. t-test was used to compare each paired treatment. The error bars represent standard error of mean (SEM) of biological replicates each represented by a data point. ns, not significant; ***, *p* < 0.001; ****, *p* < 0.0001.

Using *GFP* as a reporter gene, we further assessed direct interactions of miR-34 with the three genes whose expression was inhibited by miR-34. For this, the predicted target sequences from the three genes were cloned downstream of the *GFP* gene in the pIZ vector (Fig. S1), which were co-transfected into Sf9 cells together with the miR-34 mimic. The transcript levels of *GFP* were measured as a proxy to determine the interaction of miR-34 with the target sequences. In all the instances, except one of the two target sites in *FALDH*, application of the miRNA mimic led to significant reductions in the *GFP* transcript levels, suggesting direct interactions of miR-34 with the target sequences (Fig. 2).

**Figure 3:**
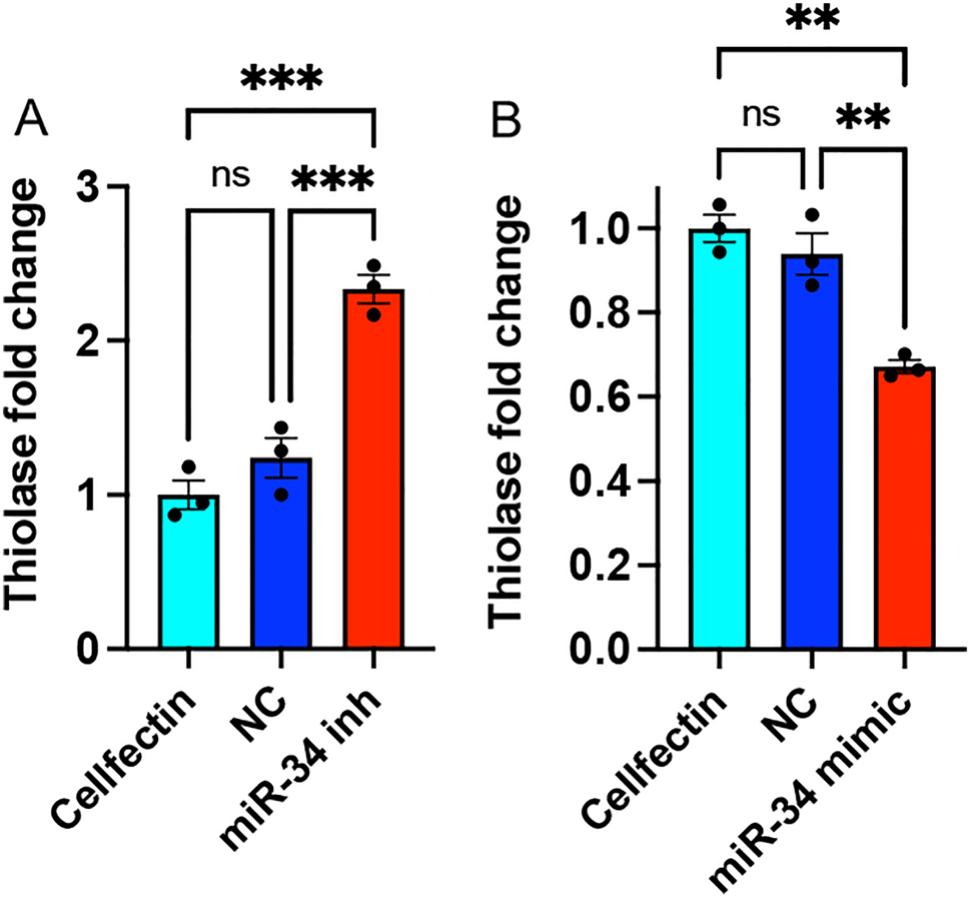
Effects of manipulation of miR-34 levels on *thiolase* transcripts in Aag2 cells. (**A**) Aag2 cells were transfected with Cellfectin, negative control (NC) inhibitor, or miR-34 inhibitor. *Thiolase* transcript levels were quantified by RT-qPCR. (**B**) Aag2 cells were transfected with Cellfectin, negative control (NC) mimic, or miR-34 mimic. *Thiolase* transcript levels were quantified by RT-qPCR. One-way ANOVA with Tukey’s post hoc analysis was used to compare the treatments. The error bars represent standard error of mean (SEM) of biological replicates each represented by a data point. ns, not significant; **, *p* < 0.01; ***, *p* < 0.001.

Thiolase, which converts acetyl-CoA to acetoacetyl-CoA, is the first enzyme in the JH biosynthesis pathway (Fig. 1A). Consequently, we selected this gene to further verify the role of miR-34 in regulating its transcript levels by transfection of *Ae. aegypti* Aag2 cells with either a miR-34 inhibitor or a mimic. In inhibitor-transfected cells, the *thiolase* transcript levels were significantly upregulated (Fig. 3A). Conversely, the *thiolase* transcript levels were significantly downregulated in mimic-transfected cells (Fig. 3B). These results confirmed that increases and decreases in miR-34 transcript levels have opposite effects on *thiolase* mRNA changes.

### The expression patterns of aae-miR-34-5p and the JH biosynthetic genes

**Figure 4:**
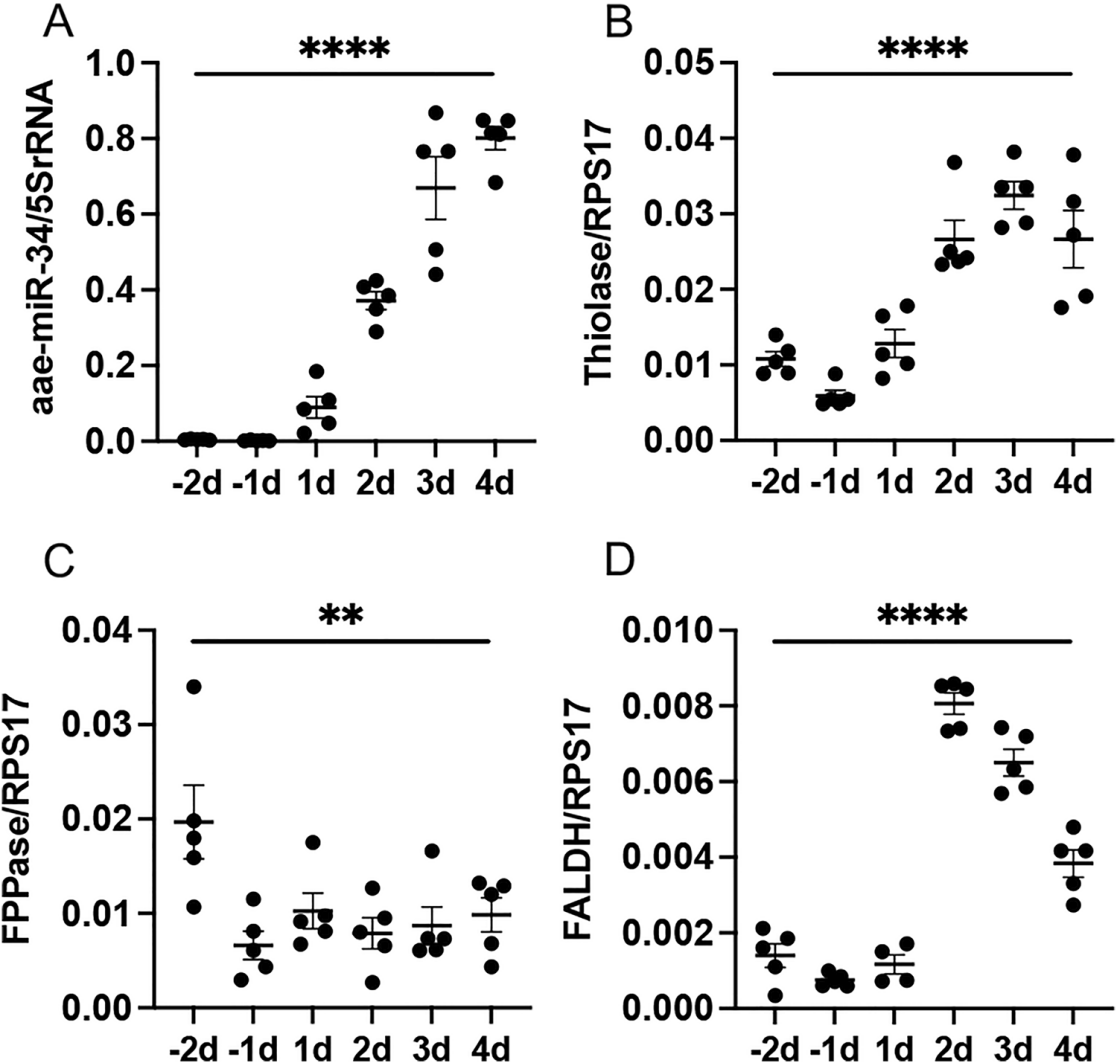
The expression patterns of miR-34 and three JH biosynthesis genes during mosquito development. Individual female pupae were collected 2 (− 2d) and 1 (− 1) day prior to adult emergence, and 1, 2, 3 and 4 days after adult emergence (1d–4d). The extracted total RNA was then subjected to RT-qPCR. (**A**) The relative expression levels of miR-34 during the developmental stages. (**B**–**D**) The relative expression levels of three genes involved in JH biosynthesis (see Fig. 1A). Each data point represents a single mosquito. One-way ANOVA was used to compare the treatments. The error bars in all the graphs represent standard error of mean (SEM) of biological replicates each represented by a data point. **, *p* < 0.01; ****, *p* < 0.0001.

We assessed the expression pattern of aae-miR-34 at the pupal and adult stages by collecting pupae (− 2 and − 1 days before adult emergence) and sugar-fed adults from day 1 to 4 after emergence. RT-qPCR analysis of RNA extracted from individual mosquitoes showed minimal detection at the pupal stage with a significant increase as mosquitoes aged, with highest expression in 4-day-old adult females (Fig. 4A).

Next, we explored the expression of the three JH biosynthetic genes studied in the same samples in which miR-34 levels were assessed. There were significant changes in the expression levels of all these genes when all ages across the developmental stages studied (Fig. 4B–D). While *FALDH* mRNA abundance showed tendencies to decrease as miR-34 abundance increased, *FPPase* and *thiolase* did not respond similarly. *FPPase* was reduced from -1 day and remained low across the days tested, and *thiolase* only showed a trend to decline from day 4.

### Effect of miR-34 manipulations on mosquito fecundity

**Figure 5:**
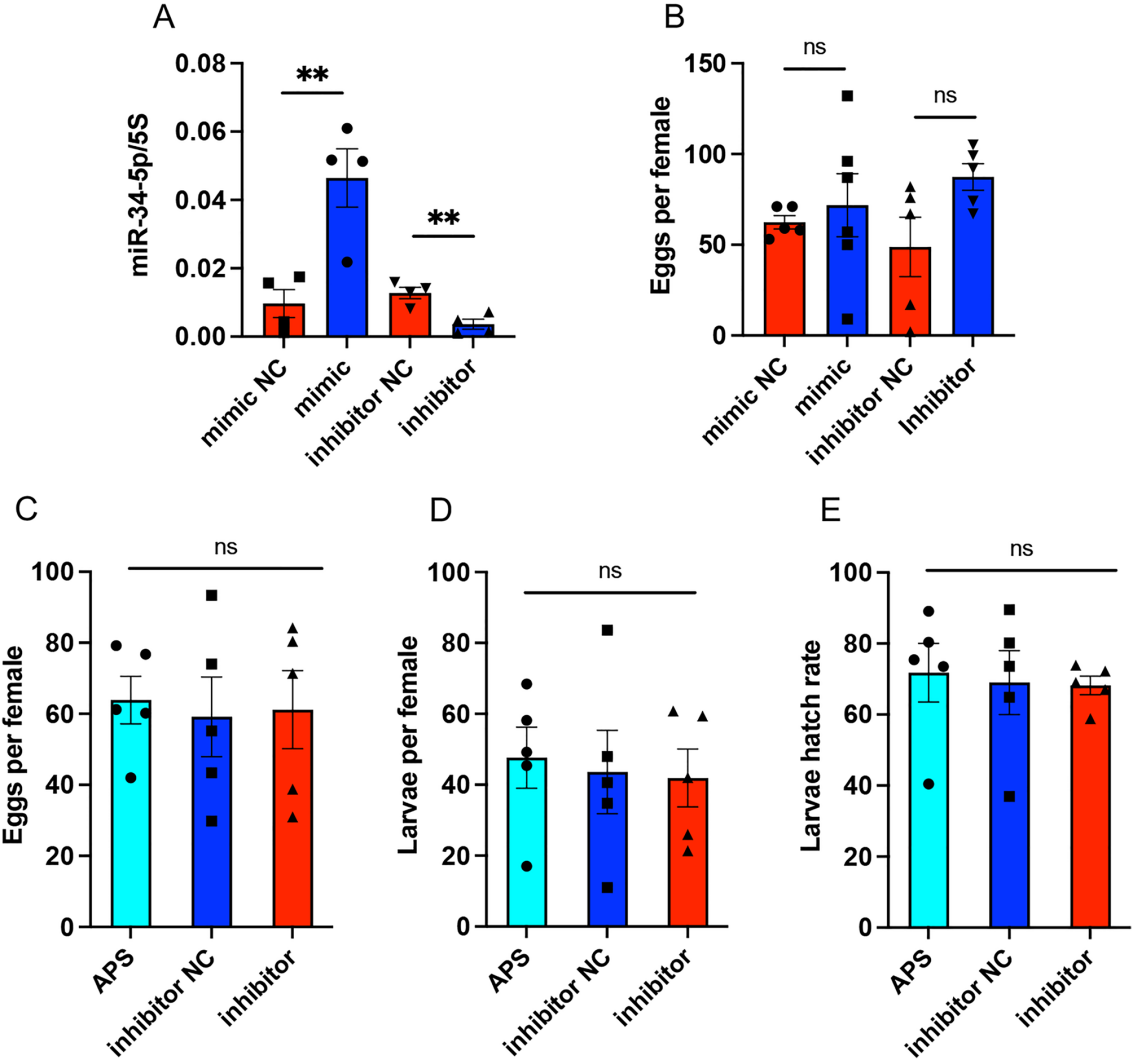
Effect of manipulation of miR-34 abundance on mosquito fecundity. One-day-old female *Ae. aegypti* mosquitoes were injected with miR-34 mimic, negative control (NC) mimic, miR-34 inhibitor, or NC inhibitor. Three days after the injection, the females were fed blood, and then allowed to lay eggs. (**A**) RT-qPCR of RNA extracted from mosquitoes three days after injection to confirm increases or decreases in the abundance of miR-34 following injection of miR-34 mimic and inhibitor, respectively. T-test was used to compare the paired samples. *p* < 0.01. (**B**) Number of eggs laid per female in the treatments. In a separate experiment, mosquitoes were injected with buffer (APS), inhibitor NC, or miR-34 inhibitor and the number of (**C**) eggs per female, (**D**) larvae per female, and (**E**) larval hatch rate were recorded. One-way ANOVA was used to compare the treatments. ns, not significant. The error bars in all the graphs represent standard error of mean (SEM) of biological replicates each represented by a data point.

To find out if inhibition or oversupply of miR-34 affects mosquito fecundity, 1-day-old female mosquitoes were injected with miR-34 mimic/inhibitor or the controls (buffer only or negative control mimic/inhibitor). Three days after the injection, the females were fed blood and allowed to lay eggs. RT-qPCR analysis of mosquitoes showed a significant increase and decrease in miR-34 abundance in mimic and inhibitor injected mosquitoes, respectively (Fig. 5A). A non-significant increase in egg number was observed in miR-34 inhibitor-injected mosquitoes. However, no significant differences were found between the number of eggs laid by mosquitoes injected with either miR-34 mimic or inhibitor (Fig. 5B). The experiment was repeated with the inhibitor, but no significant changes in the number of eggs, larvae per female, or hatching rates were observed (Fig. 5C-E).

## Discussion

Functional studies have revealed many critical roles of miRNAs amid multiple reproductive processes in female *Ae. aegypti* mosquitoes^12–15^. Insects have complex regulatory mechanisms coordinating developmental and reproductive processes, with often a mutual antagonism between the actions of JH and ecdysteroids (20E)^16^. Previous studies revealed that miRNAs regulate the JH and 20E-signalling pathways, and these two hormones reciprocally coordinate the expression of miRNAs; thus forming regulatory loops of miRNAs with the JH and 20E-signalling cascades^17^. In *Bombyx mori*, several miRNAs responded to 20E. The most strongly down-regulated miRNA was miR-34-5p, with a decrease of 104.4 folds ^18^. Conversely, treating embryonic *B. mori* (BmE) cells with a JH analogue (JHA) resulted in the upregulation of miR-34-5p^18^. In *Drosophila melanogaster*, a mutual repression exists between miR-34 expression and ecdysone signalling, with 20E and JH exhibiting opposite effects on the expression of miR-34^17,18^. miR-34 also mediates the cross regulation among the JH, 20E, and insulin pathways, to modulate wing polyphenism in brown plant hopper^19^. JH application upregulated miR-34 expression; while knocking down genes in the insulin pathway changed JH titres and miR-34 abundance; conversely, JH titers were significantly increased after treatment with agomir-34^19^.

The CA is active in sugar-fed female mosquitoes, however JH synthesis dramatically decreases after blood-feeding^1,20^. The stage-specific regulations of JH synthesis by miRNAs have not been studied in female mosquitoes. miRNA can regulate JH titres and signalling by binding to the transcripts of protein-coding genes involved in hormone biosynthesis and the downstream signalling pathways. Considering that miRNAs play key roles in the regulation of gene expression, we previously investigated changes in the miRNA profiles of CA at three developmental stages of *Ae. aegypti*, with different levels of JH synthesis: early pupa (no JH), 24 h after adult eclosion in sugar-fed females (high JH), and 24 h after blood feeding (low JH)^10^. Results showed dramatic changes in the abundance of several miRNAs when these three stages were compared.

Multiple studies proved that miR-34 plays important roles in physiological processes from lower organisms to humans (reviewed in^21^); therefore, in this follow-up study, we investigated the regulation of genes coding for a number of enzymes involved in JH biosynthesis by one of the highly expressed miRNAs in the CA, aae-miR-34-5p. Out of the 13 enzymes involved in JH biosynthesis, RNAHybrid showed potential target sequences with sequence complementarities with miR-34 in four genes (*Thiol*, *HMGR*, *FPPase*, and *FALDH*). They were selected to study the effects of injecting mosquitoes with a miR-34 inhibitor. Reducing miR-34 levels in vivo resulted in significant increases in transcript levels of three of the genes. The interactions between miR-34 and the target sequences of *Thiol*, *FPPase*, and *FALDH* were confirmed with GFP reporter constructs. The three genes studied (*Thiol*, *FPPase* and *FALDH*) showed changes in abundance that seemed, to some extent, inversely associated to changes in aae-miR-34-5p levels at different developmental stages of *Ae. aegypti* female mosquitoes, suggesting a potential regulation of these genes by the miRNA.

Our previous study showed that aae-miR-34-5p is upregulated in the CA and potentially targets FALDH. It has been previously described that the low catalytic activity of FALDH limits JH synthesis and plays a key role in the regulation of CA activity in *Ae. aegypti* females ^20,22^; interestingly, miR-34 regulates ALDH in humans, and has been implicated in cancer cell apoptosis^23^.

In our previous miRNA study^10^, we only analysed three time points: pupal stage 24 h prior to adult emergence (− 1d), 24 h after adult emergence (1d), and 24h after blood feeding. The present studies are more comprehensive, and as shown in Fig. 4A, miR-34 levels start to increase slightly in abundance at 1d, and continue to increase at the times evaluated. JH titers increase during the first days post-emergence, and after that there is a decline in synthesis that could be modulated by many factors, such as starvation and mating. While there is not a perfect correlation between the abundance of miR-34 and those of JH synthesis^20^, the observed miR-34 increases in abundance in sugar-fed females might be relevant for this complex post-emergence modulation.

Several studies have suggested roles of different miRNAs on the regulation of JH biology. In the cockroach *Blattella germanica*, RNAi experiments designed to reduce the levels of a dsRNA processing enzyme led to additional molts (supernumerary nymphs), which resembled the phenotype obtained when JH is applied ectopically to last-instar nymphs^24^. The transcript of the JH-dependent transcription factor Krüppel homolog 1 (*Kr-h1*) was upregulated following the knock down of expression of *Dicer*, the dsRNA processing enzyme^25^. In *Locusta migratoria,* miRNAs Let-7 and miR-278 bind to the *Kr-h1* mRNA, and downregulate its expression, resulting in precocious metamorphosis in nymphs, as well as a decrease in yolk production and blocked oocyte maturation^26^. Inhibition of Let-7 led to an increase of *Kr-h*1, resulting in reduced vitellogenin transcripts and an abolishment of ovarian development^26^.

In *D. melanogaster*, the overexpression of miRNA bantam decreased *JH acid methyltransferase* (*JHAMT*) expression; which reduced levels of JH III and JH bisepoxide, leading to pupal lethality. In addition, bantam overexpression resulted in malformed genitals in males, a phenotype that could be partially rescued by the application of a JH analogue^27^. Moreover, in vitro dual luciferase reporter assays and pull-down assays verified the interaction of bantam and the *JHAMT* mRNA transcript^27^.

Targeting multiple genes within the JH biosynthesis pathway by one miRNA may facilitate a fast and coordinated regulation of the genes involved in JH synthesis. Our results suggest that aae-miR-34-5p also targets *Thiolase*, the first enzyme in the JH biosynthesis pathway, involved in the conversion of acetyl-CoA to acetoacetyl-CoA. The regulation of aae-miR-34-5p titers could reduce or enhance JH biosynthesis by increasing or decreasing levels of *Thiolase* mRNA, respectively.

The ability of a miRNA to bind and regulate specific targets is influenced by the number and position of target sites within a mRNA^29^. A time-lapse study of miRNA-target interactomes in adult female *Anopheles* mosquitoes revealed dynamic miRNA regulation of gene expression in response to varying nutritional sources and physiological demands^30^. This previous study suggested the existence of varying target recognition patterns of the same miRNAs at different stages in adult mosquitoes. The alterations include small shifting of recognition sites (1–2 nt), substitution by a distal region of miRNA, and addition of supplementary pairing^28^. Similar dynamic changes in target recognition might occur in the CA of mosquitoes, and be relevant for JH biosynthesis.

Commonly, miRNAs do not totally prevent the expression of proteins, and instead only modulate the amount of their targets to maintain a specific concentration of the target protein. Many genes have their expression tuned to specific levels by vast networks of miRNAs acting in a cooperative fashion^6^. At the same time, in *D. melanogaster* the low expression of a single miRNA in the CA was not enough to seriously affect JH biosynthesis, and thus adult emergence^29^; therefore, it is not surprising that we were not able to significantly reduce mosquito fecundity by manipulating miR-34 alone.

Fine-tuning of JH biosynthetic enzymes expression by miRNAs may confer precision to the temporal coordination of hormonal titres, critical for proper egg development. miRNAs are perfectly suited to ensure exact timing and expression of genes to cope with the massive physiological demands associated with egg production. The global mapping of miRNA-target interactions contributes to our understanding of miRNA targeting specificity, providing a starting point for additional studies focused on the regulatory functions of miRNAs in the hormonal control of reproduction in mosquitoes.

### Supplementary Information

Supplementary Information.

## Data Availability

The datasets generated during and/or analysed during the current study are available from the corresponding author.
