# Supplementary material for: Investigating the role of aae-miR-34-5p in the regulation of juvenile hormone biosynthesis genes in the mosquito Aedes aegypti
Source: Sci Rep. 2023 Nov 3;13:19023. doi: 10.1038/s41598-023-46154-4 (PMC10624809; doi:10.1038/s41598-023-46154-4)
Supplement: Supplementary file 1 — Supplementary Information. [file 41598_2023_46154_MOESM1_ESM.pdf]

Investigating the role of aae-miR-34-5p in the regulation of juvenile hormone biosynthesis genes in the mosquito *Aedes aegypti*

Mazhar Hussain, Zhi Qi, Lauren M. Hedges, Marcela Nouzova, Fernando G. Noriega and Sassan Asgari

Table S1. Primers and miRNA mimics and inhibitors used in this study.

Primer	Sequence
RPS17-qF	CACTCCGAGGTCCGTGGTAT
RPS17-qR	GGACACTTCGGGCACGTAGT
Thiolase-qF	ATCGCCCAAGGAGTTCGCCG
Thiolase-qR	AGACGATAGGGAGCTCTGGAAACTT
HMGR-qF	CGTCATCTGCGCCACCGTCA
HMGR-qR	CGTCATCTGCGCCACCGTCA
PPM-Dec-qF	TCGTGTGGAAGAGTGCGTTCCAA
PPM-Dec-qR	TCCTGCATGGTGATTTTACCGAACG
FPPase-qF	TCGTTCATCCAGTCGTCAGC
FPPase-qR	CCCTGCATCCCTCAACGAAT
ALDH-qF	GGTCTGGTCGCGTAGGTAAG
ALDH-qR	GCCGAACTATCCACGTAGCA
JHAMT-qF	CCACATTGCGGACAAAATCTACGTGT
JHAMT-qR	TCCAATGGCATCCGTTCGCTGA
EPOX-qF	AGTCTGGATTTGCACCGCTGCTC
EPOX-qR	TCTGGCTGGTCGAGTTTCGATCT
GFP-qF	CCCAAGCTTCGCCACCATGGTGAGCAA
GFP-qR	CGGGGTACCCTTGTACAGCTCGTCCATGC
ALDH-1-tar-F	GG*TCTAGA*ATGACTATAGCAGACGCTG
ALDH-1-tar-R	GG*CCGCGG*GAACACAATTGCCTGCAGCC
ALDH-2-tar-F	GG*TCTAGA*GTGGTGGAATCTGCGTAAACG
ALDH-2-tar-R	GG*CCGCGG*CTACACCTAGTCCGAACAC
HMGR-tar1-F	GG*TCTAGA*GGCCGGCGAACTGAGTCTTA
HMGR-tar1-R	GG*CCGCGG*ACTCTCTATTCTTGCTGATC
HMGR-tar2-F	GG*TCTAGA*GCGCAACAAATAATGGCATG
HMGR-tar2-R	GG*CCGCGG*CTCATCGTTTGATGCTTCAA
FPPase-tar-F	GG*TCTAGA*GTCAAAGCGGTGGTGGTAGA
FPPase-tar-R	GG*CCGCGG*CGTTCTTAGCCCCACCGGTC
miR-34-qF	TGGCAGTGUGGTTAGCTGGT
5S rRNA-qF	CGCGTCAGAATGTGAACTGC
Sf9-actin-qF	TTGCCCTGAAGCCCTCTTC
Sf9-actin-qR	CTCGTGGATACCGCAAGATTC
miR-34 mimic	UGGCAGUGUGGUUAGCUGGUUG<br>ACCAGCUAACCACACUGCCAUU
Negative control mimic	UUCUCCCGAACGUGUCACGUTT<br>ACGUGACACGUUCGGAGAATT
miR-34 inhibitor	CAACCAGCUAACCACACUGCCA
Negative control inhibitor	CAGUACUUUUGUGUAGUACAA

Note: The italic residues in the target (tar) cloning primers indicate restriction sites for Xba I and Sac II.

Table S2. Predicted target genes of aae-miR-34-5p in the JH biosynthesis pathway.

<table>
<tr><th>NCBI accession number/transcript name</th><th>predicted target site</th><th>folding energy (Kcal/mol)</th><th>heteroduplex</th></tr>
<tr><td>XM_001659873.2
HMGR target 1</td><td>3381 (CDS)</td><td>-12.20</td><td>TTGCC-GTAAACCCTGGACTCCCA
  :|| |: |||| : ||| |||
GTTGGTCGATTGG--TGTGACGGT</td></tr>
<tr><td>XM_001659873.2
HMGR target 1</td><td>3477 (CDS)</td><td>-12.90</td><td>GTATAGGCACATCC-CTGTCTGCCA
     :|| || | ||||||
GTTGGTCG-ATTGGTG--TGACGGT</td></tr>
<tr><td>XM_001659873.2
HMGR target 2</td><td>4044 (3'UTR)</td><td>-15.70</td><td>GCACT--TGAACCACATTTCCA
  ||: |||||||:| |||
GTTGGTCGATTGGTGTGACGGT</td></tr>
<tr><td>XM_001654195.2
FPPase target</td><td>685 (CDS)</td><td>-16.00</td><td>AGGCCA-CCGGGCGGACGGCCA
 ::||| | : |: || ||||
GTTGGTCGATTGGTGTGACGGT</td></tr>
<tr><td>XM_001655875.2
FALDH3 target 1</td><td>521 (CDS)</td><td>-17.20</td><td>GTTCCAGTTG-CT--GCTGCTA
   ||||:|: |: :||||:|
GTTGGTCGATTGGTGTGACGGT</td></tr>
<tr><td>>XM_001655875.2
FALDH3 target 2</td><td>1337 (CDS)</td><td>-19.50</td><td>TTTGCAGTTGA-ATCACTGCCT
    |||:|:| |||||||
GTTGGTCGATTGGTGTGACGGT</td></tr>
<tr><td>XM_001657868.2
Thiolase target</td><td>224 (CDS)</td><td>-12.70</td><td>GTGGCAGC-AACACGAACTCCCA
  : |||| ||| |: ||| |||
GTTGGTCGATTG-GTGTGACGGT</td></tr>
<tr><td>XM_001657868.2
Thiolase target</td><td>316 (CDS)</td><td>-15.30</td><td>TGTGAAGCAGGCAGGCATTGCTG
     ||| :| :||:|||:
GTTGGTCGATTG-GTGTGACGGT</td></tr>
<tr><td>XM_001657868.2
Thiolase target</td><td>395 (CDS)</td><td>-15.80</td><td>GCCCCAGCAAGACAGGCGGTCA
   ||||| || :| |:||
GTTGGTCGATTGGTGTGACGGT</td></tr>
<tr><td>XM_001657868.2
Thiolase target</td><td>574 (CDS)</td><td>-17.00</td><td>TTACC--TGAAGCGCGGTGCCA
  ||| || |:|: |||||
GTTGGTCGATTGGTGTGACGGT</td></tr>
</table>

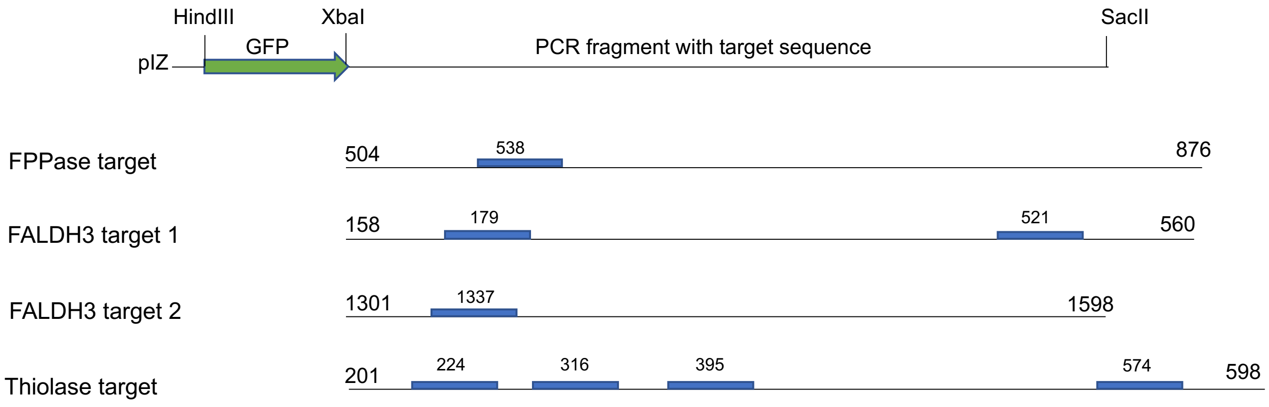

Figure S1: A diagram showing the strategy for cloning target sequences found in JH biosynthetic genes downstream of the *GFP* gene, and their positions on the corresponding transcripts. Primers were designed on nuclear coordinates shown on both ends. Blue bars show the position of the predicted target sites.
